# Expanding the causal menu: An interventionist perspective on explaining human behavioural evolution

**DOI:** 10.1017/ehs.2024.27

**Published:** 2024-10-01

**Authors:** Ronald J. Planer, Ross Pain

**Affiliations:** 1 School of Liberal Arts, University of Wollongong, Wollongong, Australia; 2 Department of Philosophy, University of Bristol, Bristol, UK; 3 Evolution of Cultural Diversity Initiative, Australian National University, Canberra, Australia

**Keywords:** causation, interventionism, potential difference making, actual difference making, specific causation

## Abstract

Theorists of human evolution are interested in understanding major shifts in human behavioural capacities (e.g. the creation of a novel technological industry, such as the Acheulean). This task faces empirical challenges arising both from the complexity of these events and the time-depths involved. However, we also confront issues of a more philosophical nature, such as how to best think about causation and explanation. This article considers such fundamental questions from the perspective of a prominent theory of causation in the philosophy of science literature, namely, the *interventionist theory of causation*. A signature feature of this framework is its recognition of a family of distinct types of causes. We set out several of these causal notions and show how they can contribute to explaining transitions in human behavioural complexity. We do so, first, in a preliminary way, and then in a more detailed way, taking the origins of behavioural modernity as our extended case study. We conclude by suggesting some ways in which the approach developed here might be elaborated and extended.

## Graphical abstract

**Figure:**
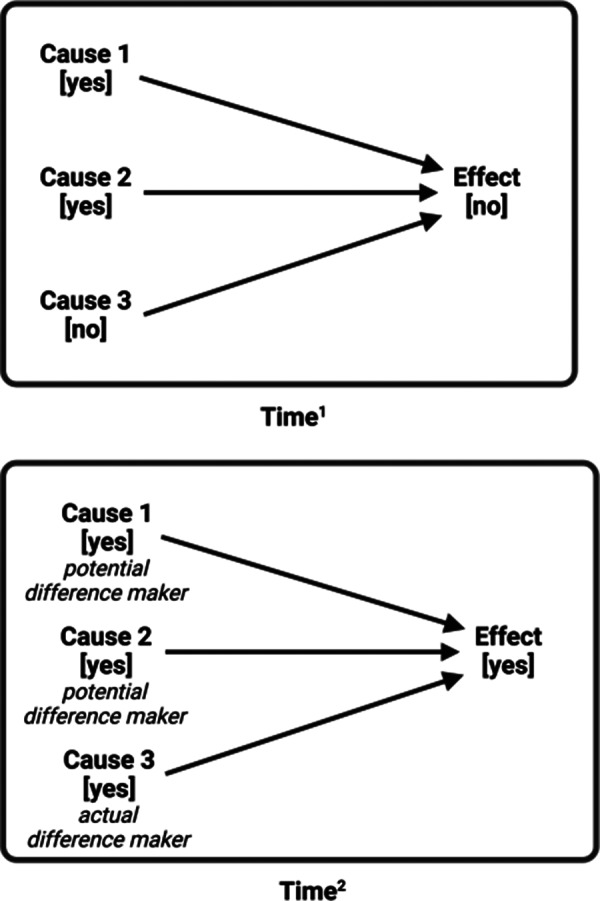

**Social media summary:** New thinking in the philosophy of causation can clarify what is at issue between competing explanations of human behavioural evolution.

## 1. Introduction

Theorists of human evolution are often interested in explaining large-scale shifts in human behavioural capacities and practices. When these changes produce major changes in hominin lifeways, they are apt to be described as ‘key transitions’ in human behavioural evolution. Paradigm examples include the invention of novel technological industries (e.g. the Acheulean) and food-getting techniques (e.g. big-game hunting), but also ‘symbolic’ modes of behaviour, such as bodily adornment and art. Explaining these transitions involves discovering their causes. What *caused* hominins to invent the Acheulean industry, for example? Needless to say, this is no easy task. Transitions in human behavioural evolution are typically spread out over significant portions of space and/or time, and hence tend to be complex physical and historical events. Moreover, the time-depths involved mean the data at our disposal is often both impoverished and ambiguous.

In addition to these empirical challenges, we also face more philosophical and conceptual problems. The most fundamental of these is providing an account of what it is for one event to be a genuine *cause* of another. It is fair to say the standard approach nowadays is some version of the *counterfactual account* of causation. In the causal inference literature, for example, it is assumed that C is a cause of E just in case, were C not the case, E would not have been the case, all else equal. Roughly put, the ‘all else equal’ clause here expresses the idea that E would not have occurred but for C, holding everything else about the situation fixed. This is the condition that randomised controlled experiments in clinical research attempt to model: to the extent that our sample is large enough and drawn truly at random, and to the extent that individuals have been assigned to the treatment and control group in a truly random fashion, whatever confounds exist between the two groups can be expected to largely cancel out. The result is a situation – the state of the control group – that reasonably approximates what the state of the treatment group *would* have been like had everything about their situation been held fixed except for their having received the treatment in question. Outside the lab, we can hardly expect our datasets to contain such a neat counterbalancing of comparison groups, and this includes data sourced from the archeological record. However, with the aid of formal causal inference methods, we can still make reasonable judgments about the existence and strength of causal connections based on observational data from the real world. These methods provide an alternative way of modelling the relevant counterfactual situations.

Likewise, a counterfactual account of causation is at the heart of a prominent theory of causation in the philosophy of science, namely Woodward's *interventionist theory* of causation. (NB: The term ‘interventionism’ is sometimes used in a broad way to cover both a Woodwardian approach to causation and causal inference approaches; in this article, we use the term to mean only the former.) In the first instance, interventionism conceives of (deterministic) causation as follows: two variables, *X* and *Y*, stand in a causal relation to one another just in case there are background circumstances in which it is possible to bring about a change in the value of *Y* by intervening on the value of *X*. (For the indeterministic case: an intervention on *X* causes a change in the probability distribution over *Y*.) The notion of an intervention is a specialist one. An intervention on variable *X* with respect to variable *Y* is a manipulation of the value of *X* such that, if the value of *Y* is changed, the change in *Y* only occurs through the change in *X*. The change in *Y* cannot occur via some alternative causal pathway that does not include *X*, for instance, via a common cause of *X* and *Y* (for a fuller discussion, see Woodward, 2003: 98).

There is considerable overlap but also some important differences between mainstream causal inference frameworks (e.g. directed acyclic graphs, structural equation modelling, and the Rubin causal model, aka the potential outcomes framework) and interventionism. Unfortunately, however, a general discussion of these similarities and differences is beyond the scope of this work (see, again, Woodward, 2003 for an illuminating discussion, e.g. §§2.2–2.3). Here, we simply focus attention on a signature feature of interventionism, namely, the distinctions it makes between different *types* of cause. Interventionism begins with a very minimal notion of causation. As a result, it classifies as causal a variety of relations among variables that it might at first seem counterintuitive to treat as genuine cases of causation (say, because of a relation's dependency on highly specific background circumstances). Basically, interventionism asks: *are there circumstances in which it is possible to wiggle the value one of variable by wiggling the value of another?* Interventionists see this minimalist treatment of causation as a feature of the theory rather than a bug. It provides an inclusive starting point from which a variety of more complex and demanding causal notions can be built up, with theoretically interesting relations holding between these notions.

For interventionists, this expanded menu of causal concepts is useful for advancing a range of conceptual and empirical issues in the sciences, and we strongly agree. Here our aim is to sketch how some of these concepts might be fruitfully applied to questions about transitions in human behavioural evolution. We begin by setting out a group of interventionist causal concepts that are especially handy for thinking about causal relations spread out over space and/or time (as is typically the case for transitions in behavioural evolution). Initially developed by Waters (2007) to deal with some issues in the philosophy of biology, these are the concepts of *a potential difference making cause*, *the actual difference making cause* and *an actual difference making cause*. We then introduce the notion of *causal specificity*. The notion of a specific cause has been discussed for some time in the philosophy of causation literature, although often under other descriptions (e.g. ‘influence’) and often as an account of causation *proper* (e.g. Lewis 2000), as opposed to a *type* fof causation. In more recent years, Woodward has neatly formulated the notion of specificity in interventionist terms (see, especially, Woodward, 2010). On the version of this notion that has attracted the most attention (Woodward formulates a few), a specific causal relationship is one in which *fine-grained changes* can be made to the effect variable by making *fine-grained changes* to the cause variable. In this article, we mainly focus on applications of specificity combined with actual difference-making causation, following Waters (2007), although specificity is an important causal concept in its own right. There are other interventionist causal concepts which we shall not discuss here, such as *stability* or *invariance* (the range and identity of the background circumstances over which a cause–effect link holds; see, e.g. Cartwright, 2003; Woodward, 2003, 2006), *proportionality* (the respective ‘levels’ of cause and effect, and whether these are ideally matched; see, e.g. Woodward, 2010, who builds on Yablo, 1997), and *pathway causation* (an ordered sequence of causal control processes; Ross, 2018). These are omitted not because they are irrelevant or even less relevant, but simply owing to space constraints. A discussion of these other concepts in the context of human behavioural evolution must wait for another day.

We explain the above causal concepts in some detail and apply them to debates in human behavioural evolution on a rather coarse level initially. Then, we zoom in on a particular case study, namely, the origins of behavioural modernity. This allows us to illustrate the fruitfulness of these causal notions in more detail. To be sure, our treatment of this case is still pretty abstract; however, we do not see this as problematic. To be clear from the outset: our aim is simply to make plausible the idea that these causal notions can do genuine explanatory work for us in the context of human behavioural evolution. The goal is not to reach firm empirical conclusions.

The discussion proceeds as follows. Section 2 explains potential vs. actual difference making causation. Section 3 then applies these ideas to some questions in human behavioural evolution in a preliminary way. Section 4 outlines the notion of causal specificity. Section 5 brings all of these concepts together in a discussion of behavioural modernity. Finally, Section 6 summarises, suggests some future lines of research, and concludes.

## 2. Different types of difference making

Imagine you're watching someone light a fire. They take a match out of a matchbox and strike it under a pile of wood. The wood ignites. At the start of this process, there was no fire; now there is. Finding yourself in a philosophical mood, you wonder *what caused the fire to light*?

*The striking of the match*, you think. And of course, you are right. However, are matters really so simple? Most obviously, you recall from chemistry class that fire needs oxygen to burn. Hence, you conclude that, were oxygen not present, there would be no fire. Nevertheless, you cannot shake the feeling that the striking of the match, rather than the presence of oxygen, is somehow more important. Were someone to ask you, ‘What caused the fire to light?’ and you responded, ‘the presence of oxygen’, this would be considered odd. If, however, you responded ‘the striking of the match’, this would be met with widespread acceptance. Why? What exactly is the difference between these two answers? Perhaps this reflects nothing more than an understandable bias: episodes of match striking simply grab our attention in a way that the presence of oxygen does not. After all, objectively speaking, both the striking of the match and the presence of oxygen are on par with one another, are not they?

In the philosophy of causation literature, this is known as the problem of *causal selection*. More precisely, causal selection refers to our tendency as explainers to foreground (or privilege, or elevate, etc.) one or a small number of causes of some event as the ‘true’ cause(s), and to regard other causal factors as mere ‘conditions’ for the former. The problem was first discussed by John Stuart Mill (1950[1843]), who regarded this tendency as rooted purely in concurrent pragmatic interests, and many influential philosophers since have agreed with him (e.g. Mackie, 1980). One much discussed interventionist innovation offers a novel solution to this problem. It revolves around the distinction between *potential vs. actual difference making causation* (Waters, 2007).

To see how the analysis works, let us introduce some variables in the interventionist style to represent the situation. Let *Strike* be a variable having values *yes* and *no* (we will capitalise variables in what follows; both variables and values of those variables are italicised). *Strike* being set to *yes* corresponds to the state of affairs in which the match is struck; *Strike* being set to *no* corresponds to the match *not* being struck. Next, let *Fire* be a variable, again with values *yes* and *no*, where *Fire* being set to *yes* corresponds to a state of affairs in which there is fire, and *no* to an absence of fire. The two variables stand in a causal relation to one another, as there are background conditions – indeed, plenty of them – in which we can manipulate the value of *Fire* (from *no* to *yes*) by manipulating the value of *Strike* (from *no* to *yes*):

*Strike* = *yes*, *no* → Fire = *yes, no*.

This relation underpins the idea that it was the striking of the match that caused the fire to light. However, now consider a third variable, *Oxygen*, which also takes values *yes* and *no*. The same reasoning that justifies our treating *Strike* and *Fire* as causally related applies here, i.e. there are background conditions (including the ones we imagine to actually hold in our example) in which we can manipulate the value of *Fire* by manipulating the value of *Oxygen*:

*Oxygen* = *yes*, *no* → *Fire* = *yes*, *no*.

It is in this sense, then, that it is right to think that both the match striking and the presence of oxygen are causes of the fire's lighting. However, while the two variables are on par in this respect, they differ crucially in another. Specifically, while both *Strike* and *Oxygen* are causes of *Fire*, only *one* of these variables *actually varied* in the leadup to the fire's lighting. More precisely, let *t* signify the period just before the fire was lit; at *t*, the pile of wood sits unlit in the fireplace. And now let *t*′ signify the period just as the fire lights. There is thus variation in the value of *Fire* between *t* and *t*′:

*Fire* = *no* at *t*, *Fire* = *yes* at *t′*.

Now, and this is the crucial point: if we look to our two cause variables, *Strike* and *Oxygen*, we see that it was only *Strike* that varied over the relevant timescale, that is, between *t* and *t*′, whereas oxygen was present throughout:

*Strike* = *no* at *t*, *Strike* = *yes* at *t*′.

*Oxygen* = *yes* at *t*, *Oxygen* = *yes* at *t*′.

So, while both *Strike* and *Oxygen* are causes of *Fire*, it is variation in *Strike*, and not in *Oxygen*, that explains the variation in *Fire* between *t* and *t*′.

In the terminology of Waters (2007), *Strike* was *the actual difference making cause* of *Fire*, while *Oxygen* was only a *potential difference making cause*. To be a potential difference maker with respect to some effect variable in a given context, it is enough to simply be a cause of that variable (in the interventionist sense) in that context. On any natural way of filling out our example with more details, there would be many other potential difference making causes of *Fire* in this case. The variable *Dry*, for instance, specifying whether the matchstick is dry (*yes*) or wet (*no*), is an obvious example. Just as if oxygen had not been present, the fire would not have lit, so too if the matchstick had been wet, the fire would not have lit.

Yet often multiple causes will actually vary. Then, there is no one causal factor that is *the* actual difference maker; instead, what we have is a group of actual difference makers, each of which is *an actual difference maker.* To see this, consider another case. You are watching someone attempt to light a burner on a gas stove. They turn the knob under the burner and strike a match just beneath the burner. The burner ignites. There is variation over time in the effect variable *Fire*: at *t*, there's no flame; at *t*′, there is. However, now, there is variation in not one but two causes of *Fire*: *Knob* (*on*, *off*) and *Strike* (*yes*, *no*).

(*Knob* = *off* at *t*, *on* at *t*′; *Strike* = *no* at *t*, yes at *t*′) → *Fire* = *no* at *t*, *yes* at *t*′.

In this case, both *Knob* and *Strike* are each *an actual difference making cause* of *Fire* (while *Oxygen* remains a potential difference maker).

Note that there are clearly aspects to this way of understanding causation that are relative to our interests as explainers (i.e. pragmatic considerations). Some of these aspects are simply inherited from the interventionist theory of causation. Even if the world came neatly pre-packaged into variables of the sort that appear in interventionist models (it does not), we would still face the task of selecting some variables rather than others in attempting to understand the causal structure of a target system. This includes the selection of a particular effect variable (or variables), the change(s) in which we seek to understand. *What we wish to explain in the first place depends on our interests.* Yet once all these choices are made, it is objective features of the target system that determine actual vs. potential difference making causality. Returning to our first example: the match was unstruck at *t*, and then struck at *t*′, while the presence of oxygen remained constant throughout. Identifying match striking as the cause of the fire thus picks out an objective fact about change in the structure of the world over time. From this perspective, then, our privileging match striking in an explanation of the lighting of the fire is not purely pragmatic (although our interest in actual difference making causation is). It is partly pragmatic, yes, but also partly objectively motivated.

Let us now begin to look at how these ideas can throw light on causal-explanatory issues in human evolution.

## 3. Explaining transitions in human behavioural evolution: Part A

Theorists offer a range of different types of causal explanations for transitions in human behavioural evolution which are often regarded, if only tacitly, as competing with one another. Here, we sort commonly cited causal factors into three categories to aid in general discussion, namely, biological, social and environmental, and limit our focus primarily to cultural behaviours.

### 3.1. Biological factors

From an interventionist point of view, we can abstractly represent the causal relations posited by this category of explanations as taking the form:

*Biological Factor B* = *x*, *y* → *Cultural Factor C* = *x*, *y*.

This is a kind of schema that biological explanations fill in with more concrete variables (and values).

Suppose that the cultural factor in question is, e.g. the appearance of the Acheulean handaxe in the archaeological record. Our effect variable is: *Handaxe* = *present, absent*. In terms of biological causes of this effect, a number have been offered. What is perhaps most impressive about handaxes, relative to earlier Oldowan tools, is the complexity of their design and hence the increased cognitive demands handaxes placed on their makers. Hypothesised causes have included enhanced working memory capacities (Coolidge & Wynn, 2018) and/or enhanced capacities for hierarchical cognition (Stout et al., 2021; Stout & Chaminade, 2012). In addition, some link the handaxe industry to the origins of novel social learning abilities, such as imitation learning (Arbib, 2011; Paddayya & Shipton, 2004).

Biological factors are typically envisaged by theorists as intrinsic traits of hominin minds (and/or bodies). By ‘intrinsic,’ we mean these traits do not depend on specific environmental conditions for their acquisition or development; they are *robustly developing* traits (Northcott & Pinccinini, 2018; see, also, Ariew, 1999). These traits are understood as largely genetically specified or ‘coded’ (although it would probably be better to think in terms of genetic canalisation here; Waddington, 1942); hence, their appearance is understood as the result of a genetic mutation, while their establishment at the population level is explained in terms of natural selection operating on genes. Some biological factors, including some cognitive ones (see, e.g. Heyes, 2018 on ‘cognitive gadgets’), are instead due to mechanisms of adaptive plasticity. However, here we shall have in mind the more common understanding of ‘biological factor’ in the literature (i.e. a strongly genetically canalised or channelled trait).

### 3.2. Social factors

Similarly, we can schematically represent social causes in interventionist terms like this:

*Social Factor S* = *x*, *y* → *Cultural Factor C* = *x, y*.

In this category are causes relating social dynamics within and/or between hominin social groups to cultural changes. Such factors are typically envisaged as capable of undergoing change independently of changes to hominins’ intrinsic biological (including cognitive) traits: holding the latter traits fixed at a time, hominin social networks can expand, contract, change their internal composition, etc. (although these changes may produce downstream biological changes). The highly influential demographic models that link hominin cultural complexity to features of social learning networks (in particular, their effective size) squarely fit in this category (e.g. Powell et al., 2009; Premo & Kuhn, 2010). So do models that emphasise not just the impact of population size, but also the unique ways in which factors like migration and meta-band structure influence cultural innovation and the spread of innovations (see, especially, Sterelny, 2021a, 2021b). A different although related line of thought that goes here connects the establishment of cooperative breeding to increases in cultural complexity. Cooperative breeding increases the social complexity of hominin lives in a variety of ways, including some that directly bear upon social learning. It has been plausibly argued, for example, that cooperative breeding provides learners with a larger pool of tolerant, in-group models to learn from (as opposed to, say, just one's mother and/or siblings; Burkart et al., 2009; Burkart & van Schaik, 2016; Hrdy, 2011).

### 3.3. Environmental factors

Finally, we can think of environmental explanations as taking the form:

*Environmental Factor E* = *x*, *y* → *Cultural Factor C* = *x*, *y*.

For example, climatic instability both at and over time has been used to explain transitions in human behavioural evolution, including cultural complexity (e.g. Richerson & Boyd, 2013; Shultziner et al., 2010). Another highly influential idea has been that the *risk* associated with particular environments promotes increases in cultural complexity (Collard et al., 2005, 2013; Collard et al., 2016; Torrence, 1983, 1989, 2001). Environments differ with respect to the risks (e.g. risks of resource failure) that they pose, sometimes sharply. According to this hypothesis, high risk of resource failure selects for a more complex and diverse tool kit. This is because, in such environments, the costs of a technological misfire tend to be dire. As such, foragers are expected to develop tools that are more specialised and (hence) more reliable; tools that better mitigate risk. Specialised tools, in turn, tend to be more internally complex (i.e. have a greater number of functional parts) and more complex to manufacture.

### 3.4. Actual vs. potential difference making causes of transitions in behavioural complexity

How do these various explanations look from the interventionist perspective outlined above? More specifically, how and when do these explanations compete? And on what grounds do they compete, when they do?

As a general rule, we think that factors from all three of the above categories – the biological, the social and the environmental – are going to be causally relevant to understanding major transitions in human behavioural complexity. Yet this is probably so only in a minimal sense, namely: there will be instances of each type of factor that indeed serve as *causes* of the effect variable of interest; that is, as potential difference makers. To see this, let us take a step back. Suppose, again, that it is the appearance of the Acheulean handaxe (or the Levallois flaking technique, or some other impressive tool form) that we wish to explain. The rationale for thinking that biological factors feature among the causes seems to run as follows: (i) tools place various task demands on makers’ intrinsic cognitive capacities (*Intrinsic Cognition*); and (ii) agents’ intrinsic cognitive capacities depend on agents’ biological makeup (for example, gross facts about their brain size and/or organisation). Thus, in general, it is possible to intervene on the set of artefacts that an agent can reliably make by intervening in biologically realistic and relevant ways (e.g. via genetic mutation) on those agents’ brains – say, their working memory abilities – and we have every reason to believe this was true in the circumstances that actually accompanied the appearance of the handaxe in the archaeological record; in particular, the appearance of *Homo erectus* (de la Torre, 2016). In this sense, it can be truly said that it takes a mind of a particular intrinsic grade to manufacture an Acheulean handaxe. *Intrinsic cognition* is among the *bona fide* causes of *Handaxe*.

The same can be said for both social and environmental factors, in our view, although the reasons for recognising causal links between these sorts of factors and changes in cultural complexity are different. More specifically, here, the grounds for positing causal relations are provided by a mix of theoretical, modelling and empirical (e.g. ethnographic) evidence. This evidence shows there is indeed a wide range of empirically realistic circumstances in which, by intervening so as to change (e.g.) population density or (e.g.) the risks of resource failure, we can bring about change in a population's material culture. All that is required for population density or resource risk to have been a genuine cause of some transition in cultural complexity is for the actual background circumstances that held at the time to have supported the relevant counterfactual relations (i.e. that the effect variable *Cultural Factor C* would have differed in its value had *Population Density* and/or *Risk* varied in its value).

However, it will by now be clear that being a cause *simpliciter*, a potential difference making cause, is one thing, while being the or a cause that actually made the difference is another. We propose this is an important way in which biological, social and environmental explanations (or two or more biological explanations, etc.) can compete in a given case, as the remarks of theorists often invite us to think (see Section 5.2), even if all these factors served as genuine causes of the transition in question. While, e.g. *Population Density* or *Risk* might have served as potential difference making causes in the context at hand, perhaps it was only *Intrinsic Cognition* that actually varied, and in so doing, actually caused the innovation of the new tool type.

Now, in saying this, we of course acknowledge that we often face serious epistemological challenges in knowing which causal factors actually varied in a population and which did not. Yet even in such cases, these causal distinctions strike us as useful tools for at least specifying ideal situations which we can then, hopefully, judge real-life cases as approximating to a greater or lesser degree. To the extent that we have reason to think only one of several causal factors under consideration actually varied (perhaps only because this is the simplest hypothesis in light of the evidence we *do* have), then this fact is probably worth marking out in some way. And the natural way to do this is by granting that factor some kind of place of prominence in our explanation of the transition. Structurally speaking, this is no different from focusing attention on the striking of the match in our first toy example in the last section. Like that case, the grounds for doing so, for elevating match striking, are at least partly objective, or put differently, not merely reflective of our interests as explainers. Once theorists agree on the effect they wish to explain (which will include specifying the temporal and/or spatial stages across which there is actual variation), and on the broader set of variables that are to be searched among for causes of this effect, it is objective facts about the structure of the world that determine which factor(s) actually made the difference to the effect in question.

The flip side of this is that a *failure* to distinguish between potential vs. actual difference making causation can lead to the appearance of conflict or competition when in fact there is none, or at least need not be any. Perhaps the most obvious way this can occur is where a theorist who is interested merely in establishing that some factor was a cause (i.e. a potential difference maker) of a transition is taken by others to be claiming that this factor actually varied in the relevant population and in so doing actually produced the effect of interest.

We now introduce another interventionist causal notion that we think has useful applications to debates in human behavioural evolution and related fields.

## 4. Specific causation

Intuitively, the influence or power a cause variable has over an effect variable can be more or less *specific*. The idea of specificity is often expressed in terms of ‘fine-tuning.’ Specific (or highly specific) causes are ones whose value you can fine-tune, and in so doing, fine-tune the value of the effect variable. In contrast, non-specific causes operate in a switch-like fashion: you can change the value of the effect variable by intervening on the value of the cause, but you cannot modulate the value of the effect variable in a fine-grained way by modulating the value of the cause variable.

**Figure 1.**
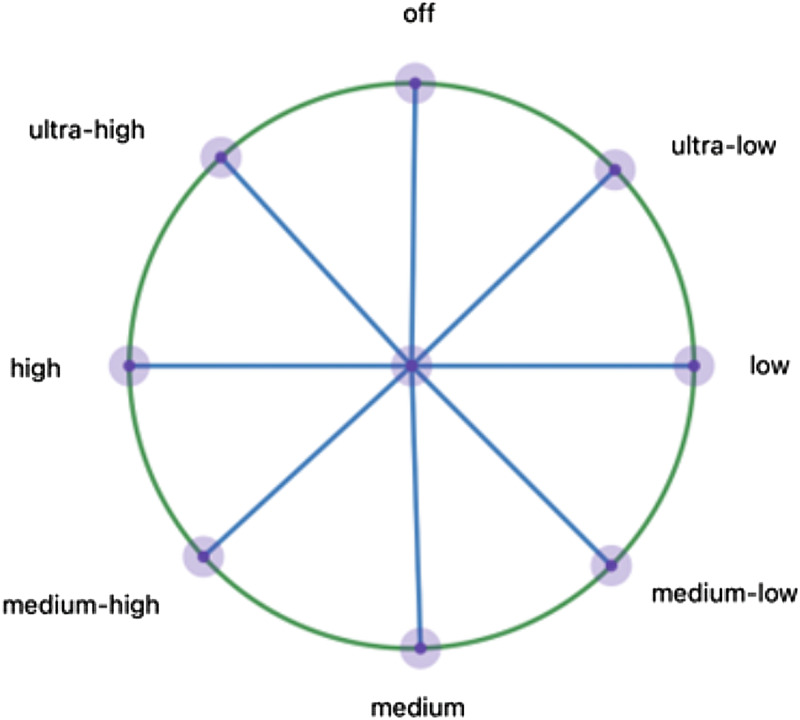
A geometric (a) and numeric (b) specification of value correspondences for Knob and Fire.

A simple adaptation of one of our above examples can be used to illustrate causal specificity. Recall our example involving the burner on the gas stove. Let us now tweak the example. As we originally described this case, the variable *Knob* had just two values: *on* and *off*. Let us now enrich the value space of this variable as follows: the knob has an off setting corresponding to 0° of rotation; an ultra-low setting corresponding to 45° rotation, a low setting corresponding to 90° rotation, and so on (see Figure 1). The value space of the variable thus looks like this:

*Knob* = *0°, 45°, 90*°, *…, 315°*.

Similarly, let us also enrich our description of the burner's state. The flame can be in a variety of states: *absent*, *ultra-low*, *low*, *medium-low*, and so on:

*Fire* = *absent*, *ultra-low*, *low*, …, *ultra-high*.

Here, we say that there is a *specific causal relation* between *Knob* and *Fire* (Woodward, 2003, 2010). For not only is it possible to change the value of *Fire* by intervening on *Knob*; it is also possible to *fine-tune* the value of *Fire* by *fine-tuning* the value of *Knob*.

This, then, is the essence of causal specificity from an interventionist perspective. While the notion of specificity is explanatorily important in its own right, here we focus on specificity combined with actual difference making causation (as in Waters, 2007). To be a specific actual difference maker, it is not enough to be a specific cause *and* an actual difference maker. Put differently, we might say that a cause can be a specific cause of some effect (in the abstract) without exercising specific influence over the effect in some actual situation. This can occur in two ways. First, a cause may be a specific cause of some effect in some background conditions, but not in those that actually obtain in the context under consideration. (A cause can be a specific cause relative to one set of background conditions but not another.) This is compatible with the cause actually varying in a fine-grained way yet nonetheless failing to serve as a specific cause in the case at hand. The conditions that are conducive to the cause functioning in a specific manner are not actually in place. Second, the background conditions that hold in a given case may be such that, *were* the cause to vary in a specific way, the effect variable would likewise vary in a specific way. And yet, as a matter of actual fact, the cause does not vary in a specific way. It might vary, but only between two values over the relevant temporal/spatial frame (hence, not in a fine-grained way). Or it might not vary at all. To be a specific actual difference maker, then, it is necessary that the effect variable *actually* vary in a (more or less) specific way, that the cause variable likewise *actually* vary in a (more or less) specific way, and that the specific variation in the effect be at least (partially) counterfactually dependent on the variation in the cause.

So, for example, suppose we were to observe the variable *Fire* to vary over time as follows:

*Fire* = *absent* at *t*;

*Fire* = *medium-low* at *t* + *n*;

*Fire* = *high* at *t + n + m* (for *n*, *m* > 0).

And now suppose that it is this variation in *Fire* over time we wish to explain. Consider, then, the variables: *Oxygen*, *Strike* and *Knob*. *Oxygen* is a cause of *Fire*, but (as before) it is only a potential difference making cause; it does not actually vary over the appropriate timescale. In contrast, not only are *Strike* and *Knob* also causes of *Fire*, but both *actually vary* over the appropriate timescale, and so both count as actual difference makers of variation in *Fire*. However, only one of these actual difference makers, namely *Knob*, is a *specific* cause of variation in *Fire*. The striking of the match, in contrast, acts like a binary switch with respect to *Fire*. Yes, by intervening on *Strike*, one can change the value of *Fire*, but what one cannot do is modulate the value of *Fire* in a fine-grained way.

When confronted by an effect variable that shows fine-grained variation in some population, theorists are often inclined to search for actual specific difference maker(s) of said variation (see Woodward, 2010 for a discussion). Even if there are other actual difference makers, specific ones may be intuitively regarded as more ‘important’ or more ‘interesting’. This tendency is most evident in cases where we seek the ability as human agents to possess fine-grained control over the effect variable (e.g. in clinical contexts), although the tendency is by no means limited to such contexts.

As with the distinction between potential vs. actual causation, then, the desire to know the specific cause(s) of some finely varying effect variable is, clearly, pragmatic in nature. However, a variable's *being* a specific actual difference maker (or a simply a specific difference maker, for that matter) of some effect variable of interest is an objective feature of the cause–effect link.

## 5. Explaining transitions in human behavioural evolution: Part B

How can the concept of specific actual difference making contribute to debates in human behavioural evolution? To begin, we note that the social and environmental factors commonly cited to explain cultural complexity – for example, population density and risk – are paradigm cases of specific causes in the above sense: (i) each is conceived of as a many-valued cause variable; and (ii) background conditions (which are often only implicitly specified) exist in which the cultural complexity of a group (as measured, for example, by the number of tools they possess, or the complexity of individual tools) can be turned up or down by turning up or down the value of these cause variables.

The situation with respect to causal specificity in the biological case is more complex. Some biological causes of cultural complexity are standardly conceived of in non-specific terms. A clear example is the possession of shared intentionality (e.g. Tomasello et al., 2005). A more complicated example is so-called *know-how copying*, a form of social learning encompassing, but not limited to, imitation learning (e.g. Bandini et al., 2020; Bandini & Tennie, 2018; Tennie, 2023). Know-how copying is generally treated as a capacity agents either have or do not have, with its presence or absence being taken to explain the type of culture observed on the part of some group. Debate continues, for example, as to whether any other great ape has the capacity to genuinely copy each other's manual behaviours (for a recent overview, see Whiten (2022), and responses therein). Yet at the same time, one might think it natural to carve up know-how copying into more and less error-prone forms (and, in any case, it is easy to make sense of an agent being more or less *disposed* to copy). Other biological causes are similarly or even more open to interpretation. For example, consider the notion of ‘cognitive fluidity’ (Mithen, 1996). The cognitively fluid mind is one in which all the mind's (previously informationally isolated) ‘modules’ can talk to one another. A fluid mind can seamlessly weave together information from its ‘naïve biology’ and ‘naïve physics’ modules, so as to create, for example, composite tools featuring both organic (wood, bone) and inorganic (stone) materials. While it is very natural indeed to imagine fluidity coming in degrees, that is not how the idea has generally been developed in the literature. Rather, Mithen and others have treated fluidity as an all-or-nothing intrinsic cognitive trait explained by the evolution of complex syntactic forms of language, itself often conceived of as an all-or-nothing trait, arising owing to a sudden genetic mutation (Berwick & Chomsky, 2016, 2019; Tattersall, 2016).

In contrast, other cases of biological factors are regularly understood by theorists as specific causes of cultural complexity (although they do not use this language). Paradigm examples here include working memory capacities (e.g. Wynn & Coolidge, 2004), hierarchical cognitive capacities (e.g. Stout et al., 2021; Stout & Chaminade, 2012) and orders of intentionality (e.g. Cole, 2016, 2019).

### 5.1. The origins of behavioural modernity

To illustrate these causal concepts in a more concrete way, we now zoom in on a particular transition in human cultural complexity, namely, the origins of behavioural modernity. By this, we have in mind the suite of behavioural, and specifically, cultural traits that are either unique to modern *sapiens*, or which are at least uniquely highly developed or prevalent in *sapiens*, compared with our Neanderthal and Denisovan cousins. While there remains significant debate among archaeologists in this area (see, e.g. Nowell (2010) and references therein on the important nuances in debates over the technological and symbolic differences between *sapiens* and Neanderthals in relation to behavioural modernity), this controversy mostly takes place below the level of what is relevant for our purposes. In our view, there is enough agreement regarding the existence both of an interesting set of technological and symbolic differences between *sapiens* and Neanderthals, as well as the timeline of the establishment of these differences, for the case study to be a useful and illuminating one.

As is well known, for a long-time orthodoxy held that the behaviourally modern package emerged suddenly in Europe around 50 kya in what was often referred to as an ‘explosion’ or ‘revolution’. The thought was that modern humans living in this region had rapidly evolved forms of culture on par with those of ethnographically known foragers. These *sapiens* were equipped with new and highly sophisticated technological forms – blades, composite tools, true projectile weapons – as well as elaborate symbolic forms, which were presumably used to navigate much more complex social worlds. In contrast, Neanderthals, who had lived in the same region for hundreds of thousands of years, had evolved few or none of these signature signs of modernity.

The (supposed) sudden onset of behavioural modernity in Europe was highly salient from the perspective of this early consensus, which fuelled belief in a biological cause: a chance genetic mutation had arose and rapidly spread to fixation among these *sapiens* (Coolidge & Wynn, 2018; Mellars, 2005; Mellars & Stringer, 1989). Yet in addition, archaeologists saw a tight connection between the complexity and sophistication of many of these novel cultural forms and intrinsic cognition. The manufacture of elaborate composite tools and cave paintings depicting supernatural entities required a new kind of mind. It was an intrinsic cognitive change that provided the ‘spark’ that ignited the Upper Palaeolithic ‘explosion’.

Such a sudden origins scenario for behavioural modernity is all but universally rejected nowadays. In their landmark paper, ‘The Revolution that Wasn't’, Mcbreaty and Brooks (2000) made the case that many of the elements of the behaviourally modern package – for example, microliths, body pigments, jewellery and art – instead appear in Africa tens of thousands of years earlier. Subsequent archaeological research strongly confirmed their gradualist account. More specifically, it is now widely agreed that many of these same innovations show a patchy temporal distribution: they appear in a region, last for a period, disappear from that region, and then reappear at some later time, presumably having been re-innovated by *sapiens* (Hiscock & O'Connor, 2006). Explaining this new data eventually motivated a second – and in many quarters, still dominant – wave of explanations for the onset of modernity, this time revolving around demographic factors (Boyd, 2017; Henrich, 2015; Muthukrishna & Henrich, 2016; Powell et al., 2009; Richerson & Boyd, 2008, 2013). To the extent that changes in demographic variables could explain changes in cultural complexity, this seemed like a much more plausible explanation of the transition, for it is easy to see how factors like population density could wax and wane over time.

Finally, and more recently, this demographic consensus has been strongly criticised by proponents of an environmental risk explanation (Collard et al., 2005, 2013, 2016; see in particular 2016: 2). With these archaeologists, the focus is primarily on technological complexity, but there are also views that connect resource strain and other crises to an expanded role for symbolism in *sapiens* groups (e.g. Straus, 2000). This line of thought is supported by general behavioural–ecological conditions, but more importantly, also by a range of empirical surveys examining the complexity of hunter–gatherer tool kits under varying conditions of risk. The key point is this: like population density, risk is something that can vary, not just over space, but also over time owing to shifting climactic conditions. Like population density, risk can rise and fall.

### 5.2. Applying the causal concepts

To bring the above causal tools to bear on this transition, the first thing we need is a clear specification of our effect. This can be *Behavioural Modernity*. And we will let this variable take the values *absent, partially present and fully present*. Obviously, this is a huge oversimplification, but this will be all we need to make our central points.

As it is actual variation in this variable that we want to explain, the next thing to do is clearly specify the relevant population. Here, we will just focus on time. Had behavioural modernity in fact appeared suddenly, as orthodoxy originally maintained, only two temporal stages would have been necessary, *t* (*Behavioural Modernity* = *absent*) and *t*′ (*Behavioural Modernity* = *present*). Now we know that will not do. We propose something like the pattern of variation over time in Table 1 to stand for the patchy onset of behavioural modernity.

**Table 1.** A highly simplified depiction of the patchy emergence of behavioural modernity over time

Time	Behavioural modernity
250 kya	Absent
200 kya	Partially present
150 kya	Absent
100 kya	Partially present
50 kya	Fully present

We now face three questions: (i) what are the causes (i.e. potential difference makers) of this effect; (ii) what are the actual difference making causes of this effect; and (iii) what (if any) are the specific actual difference making causes of this effect? We emphasise that our aim here is to illustrate possibilities, not defend particular answers to these questions. The latter would, among other things, require a much more empirically realistic setup than we are working with here.

#### 5.2.1. Intrinsic cognition

We agree with those cognitive archaeologists who emphasise the cognitive demands of many of the cultural forms associated with behavioural modernity. The artefacts and symbols symptomatic of modernity *really are* impressive from a cognitive point of view. We doubt, for example, that erectine minds were capable of innovating bow-and-arrow technology. Whether heidelbergensians, with their much larger brains, might have done so is a more difficult question. Perhaps, but perhaps the elaborate forms of symbolism known from caves like Chauvet were beyond their cognitive reach. The important point is that behavioural modernity depended on a sophisticated cognitive platform (e.g. modern or like-modern working memory capacities). If so, then *sapiens*’ *Intrinsic Cognition* was indeed a *bona fide* cause of behavioural modernity.

The real question, in our view, is whether intrinsic cognitive capacities *actually* varied over the relevant timeframe in a way that might explain the origins of behavioural modernity. In other words, was *Intrinsic Cognition* an actual difference making cause? For many theorists, the ‘sudden appearance’ of the behaviourally modern package in Europe between 50 and 40 kya was by far the most compelling argument for a biological (ultimately, genetic) explanation for behavioural modernity. However, with this origins scenario superseded by subsequent finds, many theorists now express strong scepticism over such an explanation. For example, Sterelny (2014) writes:

the material traces of modernity are much less stable than we would expect, if those traces are the social reflections of a distinctive and genetically canalised set of enhanced cognitive capacities. (p. 67)

He continues (in a footnote):

Of course, it would still be possible to suggest that the genetic change was necessary but not sufficient for modernity. But this would rob the explanatory strategy of its interest, both because of the lack of a positive case for the idea, and because attention would shift to identifying the extra factors, presumably to do with social complexity. (Sterelny, 2014)

In light of the concepts introduced above, we can see that talk of ‘necessary conditions’ in this context is ambiguous in an important sense. It is ambiguous between a factor's merely being a cause *simpliciter*, that is, a potential difference making cause of behavioural modernity, and its being an actual difference making cause. As we hope will by now be clear, these represent two different objective scenarios.

We view it as an open question whether *Intrinsic Cognition* was an actual difference making cause of behavioural modernity. Yet even if it was, it is clear that it could not have been *the* or even *a* specific actual difference making cause. This is true even if the specific form of *Intrinsic Cognition* that is envisaged to have played a role in the transition is itself a specific cause of cultural complexity (e.g. working memory capacity). This is for the simple reason that no Intrinsic Cognitive factor can be expected to appear, then disappear, then reappear again (etc.) over the 200 ky timescale over which behavioural modernity establishes. Such a scenario would be completely outlandish from a biological perspective (although this would be different if the cognitive capacity in question were instead *culturally constructed*, as in the sense of Heyes, 2018).

#### 5.2.2. Population density

Turn now to social factors. Our read of the literature is that many theorists agree that population density fluctuated over the last 250 ka, and that such variation, as indicated by the formal models, can explain the gradual and patchy onset of behavioural modernity. What is the positive evidence in support of the hypothesised fluctuations in population density that might drive this change? One line of evidence is this: Cieri et al. (2014) have plausibly connected changes in *sapiens* craniofacial anatomy (what they call ‘feminisation’) to increased levels of social tolerance in *sapiens* over the last 200 ka. The crucial link concerns reduced levels/effects of circulating testosterone in adults. Such increased social tolerance is plausibly understood as an effect (and possibly a cause) of increased levels of population density. However, interestingly, in this case, the pattern is not one of population density waxing and waning (as reflected in craniofacial anatomy), but of steadily being on the rise over this time period. (At present, genetic studies paint a complex, changing, and often conflicting portrait in this area: see, e.g. Li & Durbin, 2011; Schlebusch et al., 2012; Schiffles & Durbin, 2014; Bergström et al., 2021.)

So, whereas in the case of *Intrinsic Cognition* we are inclined to think that the main question is whether *Intrinsic Cognition* was an actual difference maker or simply a potential difference maker, here, we are inclined to think that the main question is whether *Population Density* was a specific actual difference maker, or simply an actual difference maker.

#### 5.2.3. Risk

**Figure 2.**
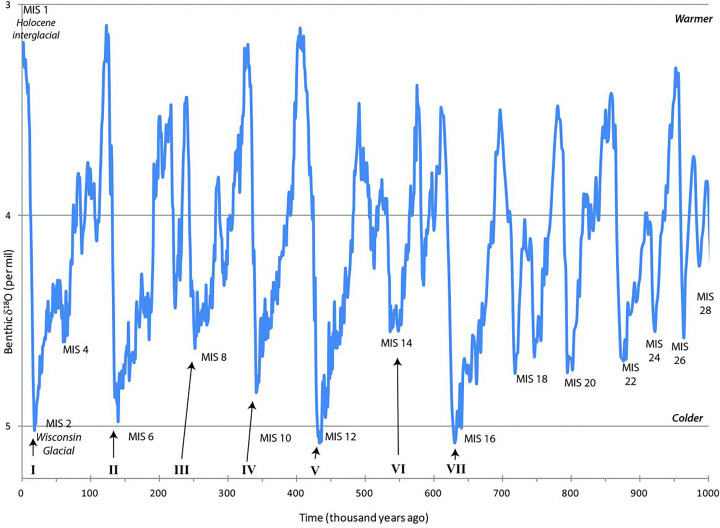
Marine Isotope Stages (over last 1000 ky). (Graph reproduced from: Illinois state government: https://iceage.museum.state.il.us/content/when-have-ice-ages-occurredImage. License: Fair Use.)

Finally, let us consider risk as a paradigm environmental factor. Beginning around 800 kya, the Earth entered a phase of marked climactic instability characterised by alternating periods of warming and cooling (see Figure 2). This pattern reached its peak over the last several hundred thousand years. With such fluctuation in climactic conditions, we would expect the risk of, e.g., resource failure in a region to likewise fluctuate over time. In Africa, colder temperatures would have led to more arid conditions, leading to a reduction in primary biomass and hence food for foragers.

The idea that a causal link obtains between risk and cultural complexity has received increasing empirical support in recent years. More specifically, a number of surveys focused on hunter–gatherer groups (as opposed to, say, farming or horticultural societies) have found that risk is a better predictor of tool-kit complexity than population density. These results are nicely summarised in Collard et al. (2016). They go on to conclude:

That more than two-thirds of the tests of the population size hypothesis that have been carried out to date do not support the hypothesis casts doubt on its use to explain patterns in the archaeological record … Given that not even a majority of studies indicate that population size is the dominant driver of cultural complexity, there are no grounds for invoking population size to explain patterns in the archaeological record. (p. 6)

We suspect that what Collard et al. have in mind by ‘dominant driver of cultural complexity’ here is quite close, if not identical to, being the specific actual difference maker of cultural complexity in a given case. We also suspect that in claiming that there are ‘no grounds’ for appealing to population density explanations of cultural complexity in the archaeological record, what these authors mean is these studies provide no reason for thinking population density is the or even a specific actual difference maker of cultural complexity. Yet at the same time, in claiming that population density is not the ‘dominant driver’, Collard et al. appear to be making room for the idea that social factors might play some role, just not an ‘important’ role. If this is correct, it would be useful for all this to be made explicit. The causal concepts and distinctions outlined here are, we think, well suited to such a theoretical task.

Here is one way all of the above types of factors might hang together in an evolutionary scenario, then. Again, we emphasise that our goal is to illustrate possibilities, rather than to defend this particular scenario.

- *Intrinsic Cognition* is a potential difference maker of *Behavioural Modernity*;
- *Population Density* is an actual difference maker of *Behavioural Modernity*; and
- *Risk* is a specific actual difference maker of *Behavioural Modernity*.

Table 2 depicts this scenario. On this hypothesis, there is no actual variation in *Intrinsic Cognition*; there is actual variation in *Population Density*, but this variation is not linked in a specific way to *Behavioral Modernity* (see the row headed by 150 kya), while finally, *Risk* serves not only as an additional actual difference making cause of *Behavioral Modernity*, but a specific one.

**Table 2.** An example hypothesis about the origins of behavioural modernity specifying different types of causes

Time	Intrinsic cognition (= potential difference maker)	Population density (= non-specific actual difference maker)	Risk (= specific actual difference maker)	Behavioural modernity
250 kya	Present	Low	Low	Absent
200 kya	Present	Medium	Medium	Partially present
150 kya	Present	Medium	Low	Absent
100 kya	Present	Medium	Medium	Partially present
50 kya	Present	High	High	Fully present

## 6. Conclusion

As an approach to causation and explanation, interventionism is distinguished by its recognition of a variety of types of cause (over and above recognising stronger and weaker causal effects). Here, we have looked at several interventionist causal notions, namely, potential difference making, actual difference making and specific causation, with the goal of exploring how these notions can benefit explanatory efforts in human behavioural evolution (and, implicitly, in human evolutionary studies more generally). At a minimum, we think that distinguishing these different roles that a causal factor can play in an explanation, and encouraging theorists to be clear about exactly what role they envisage a given causal factor to be playing, ought to help prevent theorists from talking past one another.

The approach taken here remains very basic, however. One obvious direction for future research is to consider how other core interventionist causal notions, such as *stability* or *invariance*, *proportionality* and *pathway causation*, might be fruitfully applied to debates in human behavioural evolution. However, just focusing on the causal concepts introduced here: more work is required to think through how they apply to the empirical detail of specific debates in a more fine-grained manner. For instance, it would be interesting to focus on the debate between proponents of environmental models (Collard et al., 2005, 2013, 2016) and social models (Boyd, 2017; Muthukrishna & Henrich, 2016; Powell et al., 2009) in detail. Exactly where does the conflict between them lie? And where they do conflict, can we adjudicate between these accounts? Going the other way: have avenues for fruitful synthesis been overlooked? It is noteworthy that the variation in the former models tends to be spatial, whereas the variation in the latter tends to be temporal. The evidence adduced by Collard and colleagues primarily concerns variation in forager kit across spatially disparate forager populations, whereas the models developed by proponents of the social hypothesis typically target population variation over time. We also note the potential for applications outside the *sapiens* line. For example, one might apply the framework to help impose order on and assess various hypotheses regarding Neanderthal extinction. (For a recent theoretical overview on this topic, see Currie & Meneganzin, 2022.)

However, in addition to ‘zooming in’ on debates about particular behavioural transitions, we might also ‘zoom out’. In particular, the framework developed here might be used to better understand and evaluate certain ‘single-factor explanations’ of human uniqueness. Put simply, these are accounts of the form ‘*X* made us human’, for some *X*, and are surprisingly common. Recent prominent examples include cooperative breeding (Burkart & van Schaik, 2016; Hrdy, 2011); the domestication of fire (Wrangham, 2010); shared intentionality (Tomasello, 2010); pair-bonding (Chapais, 2009); and weapons (Bingham & Souza, 2009). Clearly, none of these theorists means to claim that understanding the role played by their preferred causal factor explains the *whole* of the evolution of human uniqueness. What exactly *is* meant, then? We strongly suspect that what such theorists often have in mind is an actual difference making claim of some kind. The thought is something like this: at some point in our evolutionary past, hominins still very much fell within a range of variation considered ‘normal’ for great apes. Then something happened that put us on the human uniqueness trajectory. That trajectory itself has no doubt been highly causally complex, but perhaps its ultimate origins were not. Perhaps, that is, there was just a single actual difference making cause that kicked things off – a single cause variable that actually varied between us and other great apes at the start of this trajectory which explains why we wound up on this path and they did not. That is indeed possible (or perhaps better: it is possible that the true causal story at least approximates such a neat scenario to a considerable extent). Yet it is also possible that there never was just a single actual difference maker; that instead, humans and other great apes actually differed with respect to several causally relevant factors at the start of this process.

The bottom line is that the framework advocated in this article strikes us as well suited to clearly formulating various questions and challenges facing researchers in the field of human evolution, and in so doing, helping us to solve them. Much work, however, remains to be done in fleshing out this framework and applying it to particular empirical debates.

## Data Availability

N/A.

## Research transparency and reproducibility

N/A
